# Exogenous GR24 Alleviates Cadmium Toxicity by Reducing Cadmium Uptake in Switchgrass (*Panicum virgatum*) Seedlings

**DOI:** 10.3390/ijerph14080852

**Published:** 2017-07-29

**Authors:** Zhenglan Tai, Xinqiang Yin, Zhigang Fang, Gaoling Shi, Laiqing Lou, Qingsheng Cai

**Affiliations:** 1 College of Life Sciences, Nanjing Agricultural University, 210095 Nanjing, China; 2014116037@njau.edu.cn (Z.T.); 2014116001@njau.edu.cn (X.Y.); 2013216001@njau.edu.cn (Z.F.); loulq@njau.edu.cn (L.L.); 2 Kashghar University, 844006 Kashghar, China; 3 Provincial Key Laboratory of Agrobiology, Jiangsu Academy of Agricultural Sciences, 210014 Nanjing, China; shigaoling@jaas.ac.cn

**Keywords:** Strigolactones, cadmium, root architecture, subcellular distribution, antioxidant enzymes, endogenous GR24

## Abstract

Strigolactones (SLs) are classified into plant hormones, playing a key role as a mediator of plant growth in response to several abiotic stresses, including drought and salinity. However, the role of SLs in cadmium (Cd)-induced stress to plants is still unknown. The physiological responses of switchgrass (*Panicum virgatum*) stressed in 10 μmol L^−1^ Cd to exogenous synthetic SLs analog, GR24 were studied in hydroponics. The Cd stress significantly caused the adverse effects on plant growth and root morphology, inhibited photosynthesis, but boosted lipid peroxidation of Switchgrass seedlings. After treatment of 1 μmol L^−1^ GR24, the above adverse effects caused by Cd stress were significantly alleviated, mainly reflects in improvement of shoot biomass, relative water content, root development, chlorophyll contents, activities of typical antioxidant enzymes, nutrient uptake. The reason for exogenous GR24 alleviating cadmium toxicity might be owing to that exogenous GR24 promoted the content of endogenous SLs, increased some essential element Fe (iron), Zn (zinc), Mn (manganese) and Cu (copper) uptake and reduced cadmium uptake, accumulation and partition in shoot of switchgrass seedlings.

## 1. Introduction

Cadmium (Cd) is a known pollutant that accumulates in arable land and water resources. It is extremely toxic to plants without any beneficial function. Cd is readily taken up by the plant root and transported to other parts, threatening human health (through the food chain) as well as ecosystem safety [1,2]. Excess Cd in culture substrates causes a series of deleterious effects in plant cells. Visible morphological symptoms occur in plants, such as necrosis of leaves, browning of roots and growth inhibition [3,4]. At physiological levels, excess Cd can disturb water and nutrient uptake [5], and inhibit photosynthesis and transpiration [6]. Due to Cd toxicity in plants, plants have evolved a series of strategies to cope with excess Cd, including an active antioxidant system, vacuolar sequestration and immobilization by the cell wall [7]. Other reports indicated selective subcellular distribution of Cd in plants related with Cd detoxification [8].

Recently, GR24, a synthetic strigolactone (SL) analog, has been widely used as a plant hormone that regulates plant growth and development [9], and stimulates root hair elongation and primary root growth [10]. It was proposed that this hormone could also be involved in the biotic stresses such as drought and salinity [11]. However, little is known about the effects of SLs on plant growth under Cd stress. Whether SLs can affect Cd accumulation in plants is also unclear. Switchgrass, a warm-season C_4_ perennial grass, can be grown on marginal lands that are usually unsuitable for food crops [12,13]. This plant was found to accumulate Cd efficiently due to its high biomass production [14]. In addition, switchgrass is also regarded as an important energy crop for biofuel production. An increasing number of works focus their attention on this area. In previous studies, two switchgrass cultivars (‘Alamo’ and ‘Blackwell’) were evaluated for tolerance in Cd-contaminated soils, and ‘Alamo’ showed better performance [15]. During the past years, many approaches have been conducted to mitigate Cd toxicity and stimulate plant growth by applying exogenous materials such as silicon and malic acids as a method to develop plants in Cd-contaminated sites [16,17]. An important concern is to understand mechanisms of alleviation of Cd toxicity in plants by application of exogenous agents.

Based on above mentioned, ‘Alamo’ (lowland type) was selected as the material in this study. The objectives of the present study were to investigate the Cd phytotoxicity and uptake in switchgrass plants exposed to Cd individually or simultaneously with exogenous GR24. The parameters were with respect to plant growth, photosynthesis, micronutrient uptake, and the activity of antioxidant enzyme. The study reported in this paper is mainly focused on the mechanisms by which GR24 enhances switchgrass plant tolerance to Cd toxicity. This strategy may be used in bringing marginal lands affected by heavy metals under cultivation to meet the needs of future generations.

## 2. Materials and Methods 

### 2.1. Plant Culture and Experimental Treatments

The seeds of ‘*Alamo*’ (lowland type) were used in this study. *Alamo* plants were cultured as described by Liu [14]. Briefly, the plants were grown in half-modified Hoagland solution (containing 5 mmol L^−1^ Ca[NO_3_]_2_, 5 mmol L^−1^ KNO_3_, 1 mmol L^−1^ KH_2_PO_4_, 2 mmol L^−1^ MgSO_4_, 25 μmol L^−1^ Fe-EDTA-Na_2_, 50 μmol L^−1^ H_3_BO_3_, 0.3 μmol L^−1^ CuSO_4_·5H_2_O, 4.5 μmol L^−1^ MnSO_4_·H_2_O, 3.8 μmol L^−1^ ZnSO_4_·7H_2_O and 0.1 μmol L^−1^ [NH_4_]_6_Mo_7_O_24_·4H_2_O). The pH of nutrient solution was adjusted to 5.8 with 1 mol L^−1^ NaOH and the solution was renewed every three days. The seedlings were placed in a growth chamber with a photoperiod of 12 h light/12 h dark and a light intensity of 350 μmol m^−2^ s^−1^. The light/dark temperature was set at 30 °C/25 °C with relative humidity at 70–75%. After a 2-week acclimatization phase, Cd (CdCl_2_·2.5H_2_O, analytical grade) at a concentration of 10 μmol L^−1^ either with or without 1 μmol L^−1^ GR24 was added into nutrient solution. Thus, the treatments were as follows: The seedlings with no Cd and GR24 formed the control group (CK), plants treated only with 1 μmol L^−1^ GR24 group formed the GR24-treated group (GR24), plants exposed to 10 μmol L^−1^ Cd formed the Cd-treated group (Cd) and plants treated with 10 μmol L^−1^ Cd and 1 μmol L^−1^ GR24 formed the Cd + GR24-treated group (Cd + GR24). Three replicates were used for each treatment.

### 2.2. Determination of Plant Biomass and Relative Water Content 

After 2 weeks of treatment, plants were harvested. Roots were immersed in 20 mmol L^−1^ EDTA for 20 min to remove root surfaces with metal ions, and rinsed thoroughly with distilled water. The fresh samples were separated into roots and shoots and weighed (fresh weight, FW). The shoots were placed immediately in distilled water for 24 h in the dark, then the excess water was gently removed with paper to determine the turgid weight (TW). For dry weight (DW) estimation, the shoots and roots were dried at 70 °C for 48 h. Finally, relative turgidity (TW) and root to shoot measurements were determined using the following formula:

(1) $$\mathrm{Relative}\;\mathrm{turgid}\;\mathrm{content}\;\left(\text{\%}\right)=\frac{\mathrm{ShootFW}-\mathrm{ShootDW}}{\;\mathrm{ShootTW}-\mathrm{ShootDW}}\;\times 100$$

### 2.3. Estimation of Root Morphology

Root morphology was determined according to the method of de Graaff [18]. Briefly, three roots attached on the intact plants were selected randomly from each treatment, then rinsed with distilled water. Then, root morphological parameters, including total root length (RL), root diameter (RD), surface area (RA) and root volume (RV) were determined by using root scan apparatus (Epson, UE-88) equipped with WinRHIZO 2009 software (Quebec, QC, Canada). In order to understand the root morphological parameters, differences in size-class distribution in diameter of root systems were determined. Thus, roots were divided into three segments at intervals of 0.4 mm, i.e., 0 (root tip), 0.4 (very fine roots), 0.4–0.8 mm, and larger than 0.8 mm (coarser roots) from the root tip to the base. In separated root segments, the RL, RA, number of root tips (RT) and RV were also analyzed by WinRHIZO software as mentioned above.

### 2.4. Determination of Cd, Subcellular Distribution and Microelement Contents

The subcellular distribution analysis was conducted according to the method of Lai [19]. Plant samples were digested with 5 mL HNO_3_, according to the method of Ma [20]. Cd and microelements such as zinc (Zn), iron (Fe), manganese (Mn) and copper (Cu) were determined by inductively coupled plasma optical emission spectrometer (ICP-OES, Optima 2100DV, San Jose, CA, USA).

### 2.5. Determination of Photosynthetic Pigment and Photosynthetic Gas Exchange

The chlorophyll content was determined according to the method of Lichtenthaler [21]. Photosynthetic gas exchange parameters were measured according to Zhang et al. [22]. Samples of the mature (third) leaves were used to estimate net photosynthetic rate (Pn), stomatal conductance (Gs), intercellular CO_2_ concentration (Ci), and transpiration rate (Tr), from 8:30 to 11:30 a.m. using an LI-6400 system (LiCor, Lincoln, NE, USA) equipped with an LED light source.

### 2.6. Estimation of Lipid Peroxidation and the Antioxidant Enzyme Activities

The level of lipid peroxidation was estimated with 2-thiobarbituric acid (TBA) reactive metabolites chiefly malondialdehyde (MDA) as described by Li [23]. Enzyme solutions for activity assays were prepared according to the method of Zhang et al. [22]. Frozen leaf and root segments (0.5 g) were ground to a powder using a mortar under liquid N_2_. The homogenate was centrifuged at 12,000× *g* for 20 min at 4 °C and the supernatant was used for the enzyme extract for assays of catalase (CAT), peroxidase (POD) and superoxide dismutase (SOD) activity. All extraction operations were performed at 4 °C. The SOD activity was assayed by monitoring the inhibition of photochemical reduction of nitro blue tetrazolium (NBT) according to the method of Li et al. [24]. The CAT activity was determined following Qiu et al. [25]. The POD activity was measured with guaiacol as the substrate in a total volume of 3 mL according the method to Liang et al. with some modifications [26]. The reaction mixture consisted of 50 mmol L^−1^ potassium phosphate buffer (pH 7.0), 0.25% guaiacol, 0.75% H_2_O_2_, and 100 µl of enzyme extract. The increase in absorbance due to the oxidation of guaiacol was measured at 470 nm for 1 min. One unit of POD activity was defined as a change in absorbance of 0.1 U min^−1^. 

### 2.7. Determination of Endogenous Strigolactones Content

Endogenous strigolactones were assayed by using ultra performance liquid chromatography coupled to tandem mass spectrometry (UPLC-MS/MS), Analyses were performed using a AB SCIEX Quattro Premier XE tandem mass spectrometer (AB SCIEX, Foster, CA, USA) equipped with an electro spray ionization(ESI) source and coupled to an Agilent UPLC system (Agilent, Foster, CA, USA)., essentially as described by López-Ráez et al. [27] with a number of modifications. Briefly, 3 g of roots or shoots was ground to a fine powder in a mortar with liquid nitrogen and mixed with 4 mL water. After 15 min, 10 mL of acetonitrile the solution was added, and high-speed homogenization (10,000 r/min) was performed for 0.5 min. Then, 3 g NaCl was added to the mixed solution, then the solution was homogenized for 0.5 min (10,000 r/min). After centrifuge (3000 g) for 15 min, the supernatant fluid was dried with a flow of nitrogen gas at 40 °C. The residue was re-extracted with 1 mL of 50% (v:v) acetonitrile. Chromatographic separation was achieved using an Acquity UPLC BEH C18 column (150 × 2.1 mm, 1.7 µm) (Waters, Milford, MA, USA), applying a water/acetonitrile equilibrated column at this solvent composition for 2.8 min. Before analysis, samples were filtered through 0.22-μm filters. 

### 2.8. Statistical Analysis 

Data are the mean values ± standard deviation (SD) of three replicates. Statistical analyses were performed using SPSS Version 20.0 (SPSS Inc., Chicago, IL, USA). Two-way ANOVA (Analysis of Variance) was performed to determine the significant differences among treatments. The differences were considered statistically significant when the *p*-value was < 0.05 according to the Duncan’s test. 

## 3. Results

### 3.1. GR24 Improved Plant Biomass and Relative Water Content of Switchgrass Seedlings under Cd Stress

The effects of Cd and GR24 on plant biomass and relative water content are shown in Table 1. Plant biomass of roots and shoots was markedly reduced under Cd stress (*p* < 0.05); a similar trend was also observed in the relative water content. However, after a supplement with GR24 to Cd-stress seedlings, the fresh weight of seedlings and relative water content were significantly increased compared with Cd alone. According to two-way ANOVA analysis, Cd, GR24, and their interaction significantly affected the plant biomass, as did the interaction between Cd and GR24 in the relative water content.

### 3.2. Effects of GR24 on Root Morphology of Switchgrass Seedlings under Cd Stress

**Figure 1:**
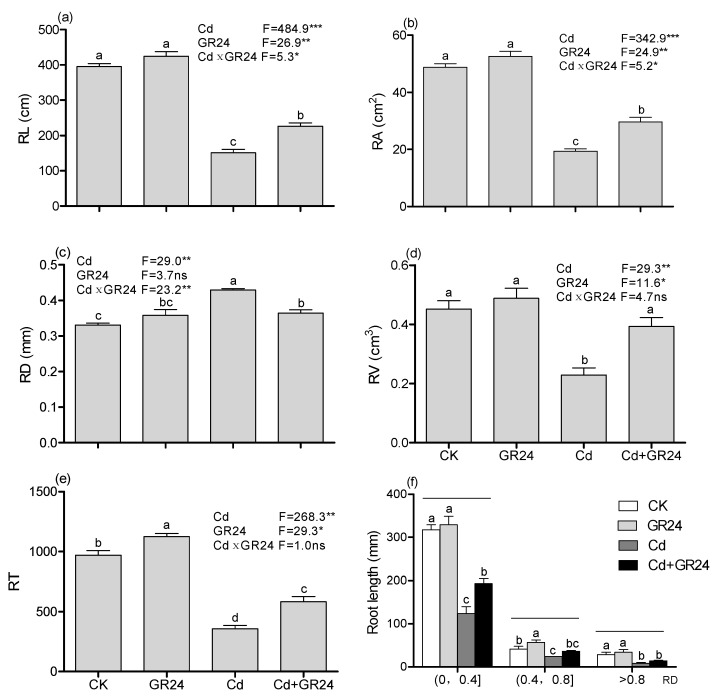
Root architecture parameters of seedlings exposed to different treatment. Values are means ± SD (*n* = 3). (**a**) RL: root length; (**b**) RD: root average diameter; (**c**) RA: root surface area; (**d**) RV: root volume; (**e**) RT: number of root tips; (**f**) root diameter classes. Different letters above the bars were significantly different (**a–e**), significant comparison under the same horizontal line (**f**) at the 0.05 level (Duncan’s test), ns: not significant, * *p* < 0.05, ** *p* < 0.01, *** *p* < 0.001.

Cd treatment alone significantly reduced the RL, RA, RV and RT. However, supplementation with GR24 in seedlings treated with Cd markedly rescued the inhibition of the RL, RA, RV and RT (Figure 1a,b,d,e). Seedlings exposed to Cd caused an increase of 29.8% in RD (*p* < 0.05) (Figure 1c), while the root length in each section (0 < RD ≤ 0.4, 0.4 < RD ≤ 0.8, RD > 0.8) was markedly decreased as compared with control (Figure 1f). By contrast, application of GR24 together with Cd increased root length by 56.9 %, 50.8%, and 64.4 % in the following section (0 < RD ≤ 0.4, 0.4 < RD ≤ 0.8, RD > 0.8), respectively (Figure 1f). Addition of GR24 in basic solution has no effect on the root morphology. According to two-way ANOVA analysis, Cd, GR24 and their interaction significantly affected the RL and RA. Cd and GR24 significantly affected the RV and RT. 

### 3.3 GR24 Reduced Cd Concentrations in Plant Tissues and at a Subcellular Level

**Figure 2:**
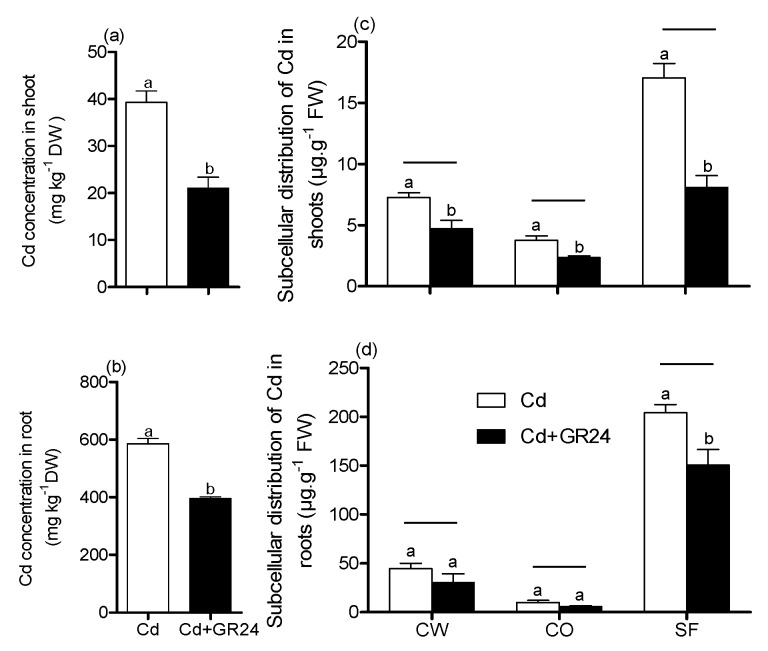
The effects of the GR24 on Cd concentration and subcellular distribution in plant tissues. Values are means ± SD (*n* = 3). CW: cell wall; CO: cell organelle; SF: solutable fraction. The dates of CK and GR24 are not shown in this Figure. Different letters above the bars were significantly different (**a**,**b**), significant differences under the same horizontal line (**c**,**d**) at *p* < 0.05. DW: dry weight; FW: fresh weight.

Cd concentration in plant tissues and subcellular level are shown in Figure 2. Apparently, the seedlings treated with GR24 in combination with Cd significantly reduced the Cd concentration in plant roots and shoots (Figure 2a,b); similar trends were observed in at the subcellular level (cell wall, cell organelle and soluble fraction) of shoots. A significant decrease only occurred in the soluble fraction of roots (Figure 2c,d). Additionally, It was found that the sequence of Cd concentration in plant cell was soluble fraction > cell wall > cell organelle.

### 3.4. Effects of GR24 on Microelement Uptake in Plant Root and Shoot

**Figure 3:**
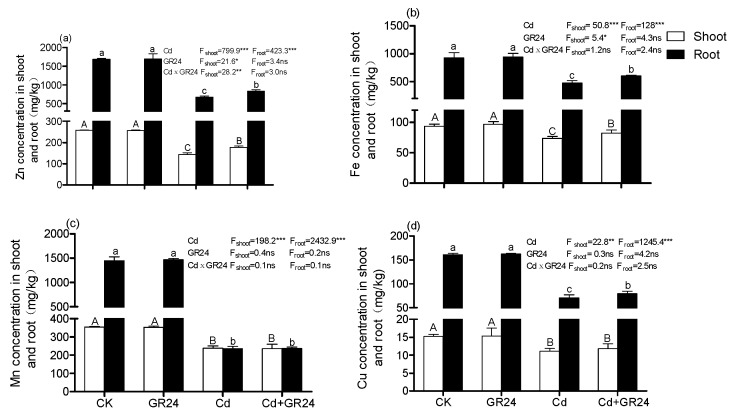
The effects of GR24 on (**a**) Zn (zinc), (**b**) Fe (iron), (**c**) Mn (manganese), and (**d**) Cu (copper) uptake in shoots and roots. Values shown as means ± SD (*n* = 3). Lower case letters represent significant differences in roots; upper-case letters represent significant differences in shoots. Different letters above the bars were significantly different at the 0.05 level (Duncan’s test), ns: not significant, * *p* < 0.05, ** *p* < 0.01, *** *p* < 0.001.

As shown in Figure 3, compared with the control, the seedlings treated with Cd caused great decreases in Zn, Fe, Mn and Cu uptake in both roots and shoots. However, supplementation with GR24 significantly increased the Zn and Fe uptake in roots and shoots, as well Cu uptake in roots. No change was observed on Mn uptake in roots and shoots when the seedlings were treated with GR24 together with Cd. A similar phenomenon was exhibited in Cu uptake in shoots. Two-way ANOVA results indicated that Cd and GR24 significantly affect Zn and Fe uptake in plant shoots, as well their interaction in Zn uptakes in shoots.

### 3.5. Effects of GR24 on Photosynthetic Pigments and Photosynthetic Gas Exchange under Cd Stress

The chlorophyll content and photosynthetic characteristics (Pn, Gs, Ci, Tr) are shown in Table 2. Compared with the control, these parameters were significantly decreased in the presence of Cd. On application of GR24 in the seedlings treated with Cd, the chlorophyll content, Pn, Gs, and Tr were significantly increased compared with the corresponding Cd-alone treatment. According to two-way ANOVA analysis, a significant interaction effect existed in photosynthetic parameters between Cd and GR24. 

### 3.6. Effects of GR24 on Lipid Peroxidation and Antioxidant Enzyme Activity under Cd Stress

Cd treatment markedly increased the lipid peroxidation product MDA content in shoots (Table 3). However, the presence of GR24 and Cd caused a significant decrease in the content of MDA in seedlings compared with Cd alone. There was no significant interaction effects between Cd and GR24 on MDA content in shoots.

The activities of SOD, POD, and CAT in shoots significantly increased under Cd stress (Table 3). Interestingly, application of GR24 in Cd-treated seedlings caused a significant increase in the activities of SOD and CAT compared with Cd stress alone. GR24 application alone significantly reduced the activities of SOD, POD and CAT as compared to Cd stress. According to two-way ANOVA analysis, Cd significantly affected the activities of SOD, POD and CAT, GR 24 significantly affected the activities of SOD and CAT. No interaction effect with respect to SOD, POD and CAT activity of shoots were observed during treatment with Cd and GR24.

### 3.7. Endogenous Strigolactones in Shoots and Roots

**Figure 4:**
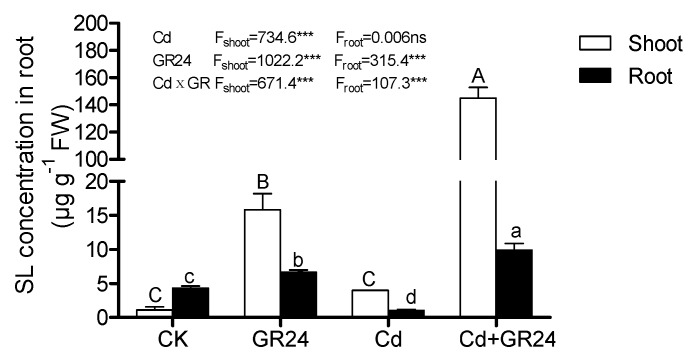
Effects of GR24 on the endogenous strigolactones in shoots and roots under Cd stress. Values are means ± SD (*n* = 3). Lower-case letters represent significant differences in roots; upper-case letters represent significant differences in shoots. Different letters above the bars indicate significant differences at the 0.05 level according to Duncan’s multiple range test. *p < 0.05, ** *p* < 0.01, *** *p* < 0.001. ns: not significant.

As shown in Figure 4, no significant changes were exhibited in the endogenous strigolactone concentration in shoots between the control and Cd treatment, while the amounts of roots in the control were significant higher in the control group compared to Cd stress alone. Application exogenous GR24 in the solution markedly increased the amounts of strigolactones, being more pronounced in the group of Cd together with GR24 treatment. According to two-way ANOVA analysis, GR24 and the interactions of Cd and GR24 significantly affected the endogenous strigolactone concentrations in shoots and roots, as did Cd in shoots.

## 4. Discussion

The present study explores the ameliorative effect of GR24 on switchgrass under Cd stress conditions. The investigation of results showed that Cd stress reduced the morphology traits (plant biomass and root morphology parameters) (Table 1 and Figure 1). The growth inhibition was observed to be associated with a Cd-induced decrease in the contents of photosynthetic pigments and the relative water content (Table 1 and Table 2); similar results were also found in the wheat seedlings under Cd stress, which is directly or indirectly related to photosynthesis and nutrient uptake [28]. Another study indicated that the growth inhibition produced by Cd could be at least partially due to Cd concentration in plant tissues and the negative effect of Cd on the photosynthesis rate, which could result in disturbances in the electron transport rates of PS1(Photosystem I) and PS2 (Photosystem II), leading to the generation of reactive oxygen species (ROS) [29]. In the present study, however, application GR24 to Cd–induced seedlings significantly increased the growth traits of switchgrass exposed to Cd stress. This could have been due in part to Cd being reduced in roots and shoot as well as at subcellular level (Figure 2). Additionally, application of GR24 recovered the Pn and Gs, which may contribute to coping with Cd by maintaining homeostasis in the electron transport rates.

It is reported that root morphology also evaluates the toxicity of plant under heavy metal stress. Previous studies showed that plant root *morphology* can be suppressed by Cd stress [30,31]. Similar results were also observed in seedlings of switchgrass under Cd stress. Kapulnik et al [32] found that SLs promoted root hair elongation through a common regulatory pathway, which is produced coordinately with ethylene in Arabidopsis. In this study, increased amounts of RL, RV, RA and RT were exhibited with supplementary GR24, which confirmed the result that the root architecture of switchgrass was regulated in the presence of SLs [31]. Another report suggested that SLs contributed to the elongation of primary roots [33]. de Graaff et al reported that the distribution of fine root (diameter < 0.5 mm) was an important trait for absorbing water, nutrients and minerals [18]. In our study, the increase in fine roots (RD < 0.4 mm) was predominate by application of GR24 under Cd stress and non-stress (Figure 1F), which may contribute to improving the uptake of water and nutrients; these results were supported by the amounts of Zn and Fe uptake in switchgrass (Figure 3).

Cadmium influences the uptake and translocation of nutrient elements in many plants. We found that Cd stress reduced the uptake of Zn, Fe, Mn and Cu in shoots and roots; the results are agreement with observations in roots by Sasaki et al [34]. Similar results were also observed in the tomato shoots when the seedlings were treated with increasing of Cd concentrations [35], indicating that Cd was responsible for mineral deficiencies or imbalances. Another mechanism of Cd toxicity is that the shortage of essential elements required for plant growth due to the competition for the same uptake systems between Cd and other divalent ions [34]. In the present study, compared with Cd stress alone, a reduction of Cd concentration and an increase of Zn and Fe in plants were exhibited after application of GR24, which confirmed the effect of competition between Cd and other mineral elements due to additional GR24. In fact, the transport systems such as the natural resistance-sssociated macrophage protein (Nramp) family and ZIP (zinc-regulated transporter) families have been identified as able to transport Fe^2+^, Zn^2+^ and Mn^2+^ as well as Cd^2+^, and are responsible for translocation of nutrient elements from roots to shoots. However, the mechanism of involving in Cd transport systems in the presence of GR24 needs to be further studied.

A decrease in chlorophyll content is the primary bioindicator of Cd phytotoxicity [28]. The well-known Cd-induced decrease in chlorophyll content has been attributed to reduced chlorophyll synthesis [36]. The investigation of this study showed that Cd-stress significantly decreased the chlorophyll contents. Exogenous GR24 could alleviate the decrease in chlorophyll content. This may be associated with GR24 acting as a positive regulator of active chlorophyll synthesis. By contrast, Woo et al reported that symptoms of leaf senescence were delayed in mutants (*max2/ore9*) with SL deficiency [37]. A similar phenotype in *dad1* (an SL-deficient mutant of petunia) was observed in petunias [38], implying that the SL acted with negative effect on synthesis of chlorophyll. Based on the above mentioned, GR24 should be involved in regulating the chlorophyll synthesis depending on treatment conditions. 

The negative impact of Cd in photosynthesis is well documented. The closing of stomata and inhibition of photosynthetic electron transport are partly responsible for an inhibition of photosynthesis [39,40]. In this study, with Cd exposure, a significant decrease in Pn was observed in switchgrass (Table 2), accompanied with the reduction of Gs. Similar trends were also observed with GR24 treatment alone. These results are affirmed in *Brassica juncea* and *Vigna radiata* by Simonova et al [41]. The decrease of Pn and gas exchange parameters may be ascribed to the stomatal closure under Cd stress and with GR24 alone. GR24 in combination with Cd increased the Pn through the alleviation of stomata closure. Concerning intracellular CO_2_ concentration (Ci), GR24 alone had no effect on switchgrass even though Pn was inhibited.

Though Cd^2+^ is not involved in redox reactions in cells, it causes production of ROS in plant [29]. Previous studies suggested that the antioxidant enzymes (SOD, POD, CAT and APX (Ascorbate peroxidase)) scavengers overproduced ROS depending on their concentrations in the cell [29]. Among the antioxidative enzymes, SOD catalyzes the conversion of O_2_^−^ to less toxic H_2_O_2_ and O_2_ and it is considered as the first line of defense against ROS. The peroxidases play an important role in scavenging H_2_O_2_ in plants [42]. We observed a marked increase in antioxidant enzyme (SOD, POD and CAT) activity and increased MDA contents in Cd treatment together with exogenous GR24 in switchgrass. The increase of SOD activity in plants is confirmed to be related with generation of O_2_^−^ in the cells and is considered as induction of protection mechanism against abiotic stresses [43]. The results from this study indicated that the enhanced antioxidative enzyme activities accelerated the elimination to H_2_O_2_ and ROS provoked by Cd stress in the presence of GR24.

Strigolactones (SLs) are multifunctional molecules recently classified as a new class of phytohormones, playing a key role as modulators of the coordinated plant development in response to abiotic stresses [44]. SLs are important for AM (Mycorrhizal fungi) symbiosis establishment [45]. AM symbiosis generally improved the growth of the host plant by facilitating water and mineral nutrient uptake [46]. Our results provided evidence that supplementation with exogenous GR24 can prompt root development and mineral nutrient uptake, as well as decreased Cd content. Zn and Fe are the key elements for photosynthesis, enhancing the activities of active leaf antioxidant enzymes SOD, CAT, and POD. Our results indicated that exogenous GR24 can alleviate Cd toxicity by promoting content of endogenous strigolactones. 

## 5. Conclusions

In conclusion, the results showed that 1 µmol L^−1^ GR24 supplementation effectively mitigated the adverse effects of Cd on switchgrass plants, and enhanced plant growth and photosynthesis. Overall, it was proposed that GR24 relieved Cd toxicity via decreasing the uptake of cadmium and improving the uptake of Zn and Fe in plants. Thus, the chlorophyll content was increased. Application of exogenous GR24 contributed to enhancing the capacity for photosynthesis by raising chlorophyll levels and mitigating Cd-induced oxidative stress by increasing antioxidant enzyme activities. 

## Figures and Tables

**Table 1:** Effects of GR24 on root and shoot biomass and root/shoot/plant relative water content of seedlings exposed to different treatments.

Variable	Fresh Weight (mg plant^−1^)	Dry Weight (mg plant^−1^)			Relative Water Content (%)	
	Root		Shoot	Root	Shoot	Root
CK	507.0 ± 16.7 ^a^		745.5 ± 51.2^a^	57.6 ± 2.9 ^a^	158.6 ± 20 ^a^	91.5 ± 2.78 ^a^
GR24	516.7 ± 98.5 ^a^		736.2.1 ± 104.9 ^a^	56 ± 5 ^a^	156.1 ± 15.7 ^a^	89.1 ± 2.2 ^a^
Cd	183.0 ± 10.9 ^b^		251.5 ± 10.2 ^c^	32.9 ± 1.4 ^b^	71.4 ± 2.4 ^c^	82.8 ± 4.8 ^b^
Cd + GR24	291.6 ± 75.3 ^b^		423.7 ± 44.1^b^	34.9 ± 5.3 ^b^	101.8 ± 13.1 ^b^	91.3 ± 0.65 ^a^
ANOVA (F value)						
Cd	45.5 ***		123.9 ***	20.6 **	34.1 ***	3.5 ns
GR24	5.4 *		5.1 *	13.4 **	8.5 *	3.1 ns
Cd × GR24	16.0 **		6.3 *	6.1 *	15.6 **	10.0 *

Data are mean ± standard deviation (SD) (*n* = 3). CK: control group. Different letters (a, b, c, d) in the same column indicate significant differences at the 0.05 level according to Duncan’s multiple range test, * *p* < 0.05, ** *p* < 0.01, *** *p* < 0.001, ns: not significant.

**Table 2:** Chlorophyll and photosynthetic gas exchange in switchgrass leaves treated with Cd and GR24

Variable	Chlorophyll Content	Photosynthetic Parameters			
		Pn (µmol CO_2_ m^−2^ s^−1^)	Gs (mol (H_2_O) m^−2^ s^−1^)	Ci (µmol (CO_2_) mol^−1^)	Tr (mmol (H_2_O) m^−2^ s^−1^)
CK	1.61 ± 0.07 ^a^	28.8 ± 1.9 ^a^	0.18 ± 0.004 ^a^	139.7 ± 4.7 ^a^	3.21 ± 0.2 ^a^
GR24	1.38 ± 0.05 ^b^	27.7 ± 1.8 ^a^	0.17 ± 0.002 ^b^	136.0 ± 3.6 ^a^	3.17 ± 0.3 ^a^
Cd	1.07 ± 0.07 ^d^	15.8 ± 0.7 ^c^	0.12 ± 0.004 ^d^	98.7 ± 2.1 ^c^	2.09 ± 0.02 ^b^
Cd + GR24	1.20 ± 0.064 ^c^	23.4 ± 0.1 ^b^	0.15 ± 0.003 ^c^	119.7 ± 2.1 ^c^	2.99 ± 0.1 ^a^
ANOVA (F value)					
Cd	97.0 ***	129.1 ***	476.6 ***	224.1 ***	38.6 ***
GR24	1.8 ns	17.8 *	63.6 ***	20.5 **	16.7 **
Cd × GR24	24.9 **	33. 1***	134.1 ***	41.5 ***	20.5 **

Values shown as means ± SD (*n* = 3). Chlorophyll content: chlorophyll a and chlorophyll b. Pn: net photosynthetic rate; Gs: stomatal conductance; Ci: intercellular CO_2_ concentration; Tr: transpiration rate. Different letters (a, b, c, d) in the same column indicate significant differences at the 0.05 level according to Duncan’s multiple range test , ns: not significant, * *p* < 0.05, ** *p* < 0.01, *** *p* < 0.001.

**Table 3:** Lipid peroxidation and antioxidant enzyme activity treated with Cd and GR24.

Variable	MDA Content (nmol g^−1^ FW)	Enzyme Activity		
		SOD (U·g^−1^ FW)	POD (U·min^−1^·g^−1^ FW)	CAT(U·min^−1^ ·g^−1^ FW)
CK	11.7 ± 5.4 ^b^	96.1 ± 5.7 ^d^	517.1 ± 15.3 ^b^	178.5 ± 5.4 ^d^
GR24	10.0 ± 0.5 ^b^	107.8 ± 3.7 ^c^	520.3 ± 16.3 ^b^	201.5 ± 7.1 ^c^
Cd	17.8 ± 1.7 ^a^	121.3 ± 1.4 ^b^	601.1 ± 39.5 ^a^	250.5 ± 3.0 ^b^
Cd+GR24	12.6 ± 3.1 ^b^	134.1 ± 1.8 ^a^	626.1 ± 43.0 ^a^	284.5 ± 10.9 ^a^
ANOVA (F value)				
Cd	16.8 ***	155.4 ***	27.7 ***	349.5 ***
GR24	10.7 *	34.8 *	0.61 ns	47.3 **
Cd × GR24	2.9 ns	0.07 ns	0.36 ns	1.8 ns

Values are means ± SD (*n* = 3). SOD: superoxide dismutase; POD: peroxidase; CAT: catalase. Different letters (a, b, c, d) in the same column indicate significant differences at the 0.05 level according to Duncan’s multiple range test, ns: not significant, * *p* < 0.05, ** *p* < 0.01, *** *p* < 0.001.
